# SHEMANCIPATION: A GROUNDED THEORY OF AFRICAN AMERICAN WOMEN’S PROCESS OF TRANSITIONING TO AGE 50

**DOI:** 10.1093/geroni/igae098.3892

**Published:** 2024-12-31

**Authors:** Lisa Bradley

**Affiliations:** Henry Ford College, Southfield, Michigan, United States

## Abstract

The study of adult development has produced numerous theories to describe participants’ experiences, processes, and related developmental milestones. Despite the number of studies found in the literature, few have explored adult development for African American women, and fewer still have explored African American women’s midlife experience. The present study, rooted in theories of intersectionality and critical gerontology, used constructivist grounded theory to investigate a discrete aspect of midlife aging for African American women, the age-50 transition. Guided by the research question, “How do African American women describe their experience of turning 50?”, eighteen semi-formal interviews were conducted with 11 African American women, age 50 to 55, which coincides with Levinson’s (1986/1996) Middle Adulthood. The women confirmed that they were born, raised, and currently lived in the United States. During data collection, constant comparison and memoing were used to evaluate the data for gaps and overlapping instances. Initial coding generated the assignment of 161 unique codes. After four rounds of coding, the three processes of Reflection, Realization, and Resolution, and the theoretical concept of “Shemancipation” emerged. Findings of the present study were partially aligned with Cohen’s (2005/2006) stages of Midlife Reevaluation and Liberation, and Howell & Beth’s (2002) midlife development stages of External Awareness and Internal Awareness stages. The findings report includes several direct quotes from the participants to intentionally amplify voices long omitted from the literature. Further study with additional cohorts was recommended, as well as an investigation of the role played by the global pandemic on introspective processes.

